# Laser Scanning Holographic Lithography for Flexible 3D Fabrication of Multi-Scale Integrated Nano-structures and Optical Biosensors

**DOI:** 10.1038/srep22294

**Published:** 2016-02-29

**Authors:** Liang (Leon) Yuan, Peter R. Herman

**Affiliations:** 1 The Edward S. Rogers Sr. Department of Electrical and Computer Engineering and Institute for Optical Sciences, University of Toronto, 10 King’s College Road, Toronto, Ontario, M5S 3G4, Canada

## Abstract

Three-dimensional (3D) periodic nanostructures underpin a promising research direction on the frontiers of nanoscience and technology to generate advanced materials for exploiting novel photonic crystal (PC) and nanofluidic functionalities. However, formation of uniform and defect-free 3D periodic structures over large areas that can further integrate into multifunctional devices has remained a major challenge. Here, we introduce a laser scanning holographic method for 3D exposure in thick photoresist that combines the unique advantages of large area 3D holographic interference lithography (HIL) with the flexible patterning of laser direct writing to form both micro- and nano-structures in a single exposure step. Phase mask interference patterns accumulated over multiple overlapping scans are shown to stitch seamlessly and form uniform 3D nanostructure with beam size scaled to small 200 μm diameter. In this way, laser scanning is presented as a facile means to embed 3D PC structure within microfluidic channels for integration into an optofluidic lab-on-chip, demonstrating a new laser HIL writing approach for creating multi-scale integrated microsystems.

The top-down approach to nano-structuring^1^ has greatly evolved over the decades to underpin today’s most important trends in science and technology. Highly resolving beams of electrons^2^, ions^3^, and photons^4^,^5^ are regularly applied in high resolution surface patterning, for example, providing the narrow 16-nm (and beyond) transistor gate widths as required in today’s commercial microchips with laser lithography^5^. Smaller 4 nm dimensions are otherwise available at much slower processing speed by direct writing with electron or ion beams^2^,^3^. An emerging opportunity here centers on new approaches that can transform rapid nano-structuring into the third dimension. Assembled layering of 2D-structures is exceptionally tedious and slow^6^ and direct 3D nano-structuring is highly challenging, as charged particles cannot propagate without scattering inside solid material. Hence, high resolution 3D patterning has been best met with optical approaches^4^,^7^ to provide advanced nanostructures that underlie omnidirectional stopbands^7^ and superprism effects^8^ in photonic crystals (PCs), negative refraction^9^ and broadband circular polarizers^10^ in metamaterials, engineered tissue growth^11^ and controlled drug release^12^ in scaffolds and carbon nano-tube composites^13^ in 3D templates.

Direct writing with tightly focused laser beams has been attractive for flexible and high resolution structuring of 3D features to sizes as small as 9 nm, representing a small fraction of the laser wavelength (~λ/90) in photoresist^4^. This patterning approach has underpinned many broadly based nanostructure applications^4^,^7^,^8^,^9^,^10^,^11^,^12^,^13^, while offering the flexibility to machine fully-3D-shaped microdevices^14^, optical waveguides embedded in 3D PC templates^15^ and optical cloaking metamaterials^16^. On the other hand, today’s highly powered lasers are highly favored in holographic interference lithography (HIL) for expanding the fabrication area and speeding the process time of 3D PC templates with resolution at one half optical wavelength^7^. Various configurations of free space and phase mask beam splitting have permitted flexible tuning of the crystal symmetry between Tetragonal (TTR)^17^, woodpile^18^,^19^ and diamond-like^20^,^21^,^22^ structures, and from simple Bravais lattices^23^,^24^,^25^ to compound^26^, chiral^27^ and quasi-crystalline structures^28^. However, traditional free-space methods for beam splitting and combining^7^,^20^,^29^ have given way to the more stable methods of beam interference with prismoids^18^,^26^, transmission gratings^30^, and proximity phase mask^17^,^31^,^32^,^33^,^34^ techniques that firmly lock together the phases of multiple diffracted beams to stabilize the 3D interference fringe pattern^17^,^18^,^19^,^26^,^29^,^30^,^31^,^32^,^33^,^34^.

Despite the advantages of rapid parallel processing, the HIL methods have been limited to static beam exposure with 3D periodic structures forming only within the area of the overlapping laser beams. Further, the areal uniformity is highly dependent on the laser beam quality. A more flexible means for embedding multi-functional components within such uniform periodic nanostructures have also been pursued by various groups^15^,^32^,^35^,^36^,^37^,^38^,^39^,^40^,^41^,^42^,^43^ towards integration of nano- and micro-structured systems^36^. In one approach, laser direct writing, when following an HIL exposure step, has enabled optical defect waveguides to be registered within the pre-existing 3D periodic structure^15^,^40^,^41^. Alternatively, multi-scale devices have been formed by multi-exposure steps of shadow and phase masks to embed 3D PCs within microfluidic channels^32^,^37^,^38^,^39^. The addition of such functionality would be more appealing if possible in a single exposure step. In another approach, a spatial light modulator^43^ has generated ~5 μm periodic structures over a small ~100 μm exposure zone. More robust and higher throughput approaches are therefore desirable in multi-scale and monolithic fabrication that can offer high speed parallel processing of 3D nano-structures over large area in a single exposure step.

In this paper, the concept of beam scanning in 2D laser projection lithography^5^ is extended to proximity phase masks by introducing direct-write laser scanning as a new hybrid direction for micro-scale structuring in 3D HIL nanopatterning. Here, for the first time, the advantage of phase-locked multi-beam interference is applied with a direct-write beam scanning to flexibly pattern and integrate 3D photonic nanostructures with microfluidic structures. The hybrid approach opens a new unexplored domain where elements of direct-writing and 3D structuring are blended for intermediate benefits of high-resolution nano-structuring with overall 2D pattern control (refer to Supplementary Fig. S1). This approach builds on the overlapping scanning exposure we first introduced in ref. 44 to overcome the prior limitation found in the uniformity and areal size of 3D PCs fabricated by static exposure^17^,^18^,^19^,^20^,^21^,^22^,^23^,^24^,^25^,^26^,^27^,^28^,^29^,^30^,^31^,^32^,^33^,^34^,^35^,^36^,^37^,^38^,^39^,^40^,^41^,^42^,^43^. This scanning approach has been further extended here to higher resolution and non-uniform beam exposure to present a wider breadth of application. Flexible exposure conditions were examined theoretically and matched with experimental demonstration in forming uniform 3D periodic templates with multi-scanned beams of various uniform and non-uniform profiles. The interference pattern of the moving diffracted beams remains phase-locked to the phase mask to permit the seamless stitching of nanostructure in thick photoresist film. Body-centered tetragonal (BCT)-like nanostructure is presented with low variance in the optical Γ-Z stopbands (∆λ_*peak*_ < 1%) across multiple overlapping exposure zones. These benefits are further scaled to small beam diameter, assessing against the challenges of small working distance as imposed by the widely diverging diffraction orders from the phase mask that lead to incomplete interference and subsequent distortion of the nano-structure motif in thick photoresist^45^. In this way, velocity controlled direct-write exposure of a single phase-mask is illustrated to embed 3D PC nanostructures flexibly within wells and microfluidic channels, with micro-scale resolution of ~200 μm. This novel laser writing method enables PC functionality in laboratory on chip devices, where waveguides, microfluidic channels and 3D PC structure are presented for on-chip fluorescence detection of low dye concentration (4 × 10^−9^ mol/ml). This top-down writing method is highly flexible in forming high-resolution patterns of 3D nanostructures through a simple phase mask that can be scaled with high power lasers to high writing speeds, attractive for opening new directions in high resolution 3D nanofabrication.

## Results

### Holographic interference lithography: static and scanning-line exposure

**Figure 1:**
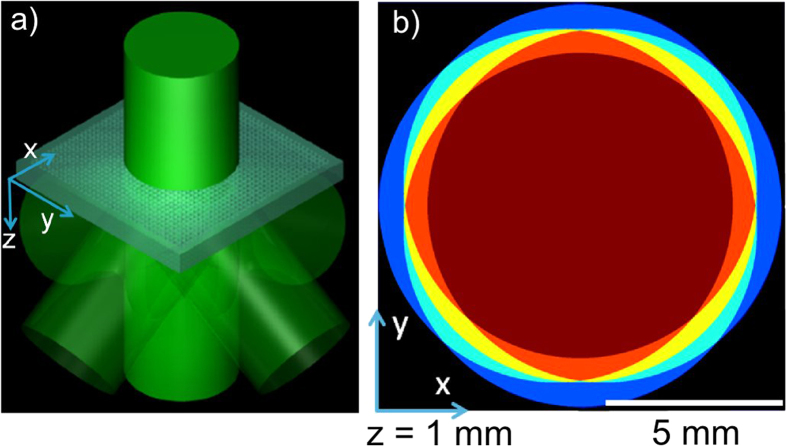
Generating five-beam interference patterns with a single 2D binary phase mask. Schematic showing (**a**) an incident laser beam of 10 mm diameter, diffracted by a phase mask into 0^th^ and four 1^st^ order interference beams and (**b**) the resultant partial separation of the beam pattern at 1 mm exposure distance indicating zones of complete beam interference (dark red) surrounded by exposure zones containing 4 (orange), 3 (yellow), 2 (cyan) and 1 (light blue) interference beams.

Conventional 3D interference lithography with a phase mask typically involves irradiating a statically-placed diffractive phase mask, such as a 2D periodic grating, with a laser beam to generate multiple transmitted beams of diffraction as shown by the 0^th^ order and four 1^st^ order beams in the schematic of Fig. 1a. The laser beam diameter defines the working distance for photoresist exposure over which all the diffraction beams will appreciably overlap and fully interfere on the opposite side of the phase mask. At an increasing exposure distance, the region of 5-beam interference (dark red) will shrink as depicted in Fig. 1b to reveal annular exposure zones of 4-beam (orange), 3-beam (yellow), 2-beam (cyan) and 1-beam (light blue) interference where lower exposure is expected together with undesirable light patterns due to missing diffraction orders as observed in ref. 45.

The present zones represent photoresist exposure of a 10 mm diameter top-hat beam through a 2D binary phase mask (570 nm period) at a 1 mm exposure distance, yielding a large uniform full interference zone of ~8.5 mm diameter surrounded by a ~2.5 mm annular exposure zone of incomplete beam interference. The annulus of incomplete interference shrinks to only ~27 μm for a closer exposure distance of 40 μm (refer to Supplementary Fig. S2a). Alternatively, reducing the beam diameter from 10 mm to 60 μm at 40 μm exposure distance leads to a significant decrease of the full five-beam interference zone from 99.0% areal overlap to 56% (refer to Supplementary Fig. S2c).

**Figure 2:**
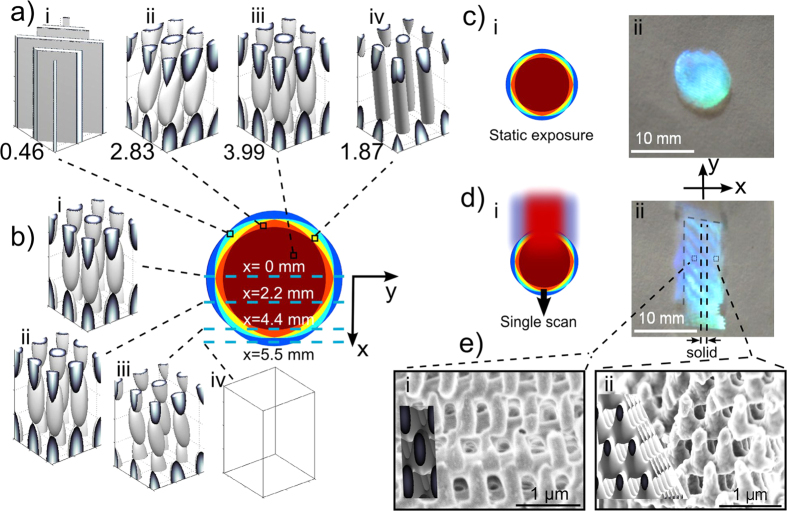
Distortions in static and scanning line exposure of binary phase mask by incomplete interference arising from missing diffraction orders. The expected beam overlap pattern (center) under the same beam conditions as in Fig. 1b and (**a**) the simulated isointensity motifs (left to right) expected in static exposure from the cyan, orange, dark red and yellow color-coded zones representing the respective 2-, 4-, 5- and 3-beam interference zones with the relative peak values of interference intensity shown below each motif, and (**b**) in single-scan exposure from center to side at *x* = 0, 2.2, 4.4 and 5.5 mm lateral offsets. Beam overlap pattern (i, left) and optical image (ii, right) of developed photoresist are shown for (**c**) static exposure and (**d**) single scan exposure. Microscopic cross-sectional SEM images of (**e**) less (i) and more open (ii) bicontinuous structures found near the center and on the side beam positions of a thick SU-8 sample, respectively, closely matching with simulated isointensity shapes (inset images). A doubling of the image (outlined by dashed line) in Fig. 2d-ii is attributed to a weak image reflection at the bottom surface of the glass substrate. The large-period fringes in Fig. 2d-ii arise from thin-film interference in the photoresist film.

The various interference zones for the case of large diameter beam exposure (10 mm), reproduced in Fig. 2 (center), are assessed individually to determine the isointensity surface expected in the photoresist from each exposure zone. The calculated surfaces are shown in Fig. 2a, providing the predicted motif and crystal periodicities of *a* = *Λ*_*x*_ = *Λ*_*y*_ = 570 nm along the *x* and *y* axes and *c* = 1.845 μm along the *z* axis that follows the reciprocal relationship between wave vector differences, namely, 
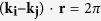
. The peak value of interference intensity (0.46 to 3.99) expected in each zone is also given, normalized to that of the incident beam. The 5-beam zone (dark red) provides a symmetric TTR or BCT-like structure with a strong interference peak intensity of 3.99 (Fig. 2a-iii). A skewed BCT-like structure and unconnected column array structures are noted when one and two diffraction orders are missing, yielding weaker intensity peaks of 2.83 (Fig. 2a-ii) and 1.87 (Fig. 2a-iv), respectively. The isointensity surface was set at a threshold intensity exposure of *I*_*th*_ = 1.2 in Fig. 2a-ii through 2*a*-iv, while a lower threshold of 0.4 was required to reveal the pattern of 1D periodic plates (Fig. 2a-i) produced in the 2-beam zone (light blue) which had a low interference peak intensity of 0.46.

To meet the objective for extending the 3D PC structure over larger area and with better uniformity, a scanning exposure of the laser beam in a straight single line (y axis) along the phase mask was investigated to determine the influence of partial exposure from the incomplete interference zones in the beam periphery. These weak exposures may distort and overshadow the ideal 3D periodic interference pattern otherwise expected from the full 5-beam interference zone. The photoresist position is locked in the proximity zone of the phase mask to ensure the interference pattern does not shift and wash out the 3D patterning during the laser scan. The isointensity surface structure predicted for the center beam exposure position (*x* = 0) is presented in Fig. 2b-i, calculated for a threshold intensity of *I*_*th_scan*_ = 1.2, and compared with structures expected at the lateral offset positions of *x* = 2.2 (Fig. 2b-ii), *x* = 4.4 (Fig. 2b-iii) and *x* = 5.5 mm (Fig. 2b-iv). The isolated voxels generated for offsets of *x* = 0 to 4.4 mm share a similar BCT-like symmetry that attests to the dominant contribution of the 5-beam interference over the weaker but distorted symmetry patterns imposed by the incomplete interference annular exposure zones. However, this 3D structure is seen (Fig. 2b) to skew and dis-connect with increasing lateral offset (from *x* = 0 to 5.5 mm) due to lower net exposure as well as due to a larger relative intensity contribution from incomplete interference zones.

An experimental comparison of the predicted nanostructure shape for static (Fig. 2a-iii) and scanning (Fig. 2b) exposure was made in 40 μm thick photoresist exposed through a 1 mm thick binary phase mask with a 10 mm diameter laser beam as in the arrangement of Fig. 1a. (refer to Methods—Photoresist preparation and development and Laser scanning holography) The photoresist substrate was gently mounted against the phase mask surface and remained stationary during the scanning exposure. Static exposure yielded the ~8 mm diameter PC structure zone shown in Fig. 2c-ii that matches with the reduced size expected for the full 5-beam interference zone as shown to scale in Fig. 2c-i (reproduced from Fig. 1b). The formation of 3D BCT-like structure here has been previously reported in ref. 46 using static exposure, yielding *a* ≈ 570 nm transverse and *c* ≈ 1.54 μm axial periods.

**Figure 3:**
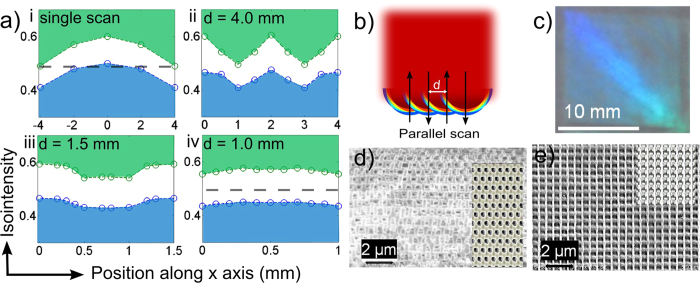
Optimizing beam overlap in parallel scanning holographic interference lithography to improve the uniformity of bicontinuous 3D PC structure. (**a**) Exposure zones predicting formation of solid photoresist (blue), bicontinuous structure (white), and underexposed photoresist (green) expected as a function of isointensity exposure level (vertical scale) against the lateral offset from the beam center applied in (i) a single scan exposure of 10 mm diameter beam and *z* = 1 mm exposure distance, and overlapping parallel scans of the same beam with scan-to-scan offsets of (ii) *d* = 4 mm, (iii) 1.5 mm and (iv) 1 mm, respectively. The parallel multi-scan approach, shown schematically in (**b**), produced a uniform 3D PC structure in SU-8 photoresist over the large area shown in the optical image (**c**), and verified by SEM imaging to yield bicontinuous BCT-like structure in both (**d**) cross sectional and (**e**) top views of the thick photoresist that matched with the simulated structures (insets in (**d,e**)).

Scanning of the laser across the same stationary phase mask arrangement at the same exposure power produced the elongated (7 mm × 18 mm) 3D PC structure in SU-8 photoresist as shown in the optical image of Fig. 2d-ii. The cross-sectional SEM views shown in Fig. 2e for two different scanning offset positions of *x* = −2 (Fig. 2e-i) and *x* = 3 mm (Fig. 2e-ii) reveal differing filling fractions, but similar formation of BCT-like structure. The photoresist structures, overlaid as insets in Fig. 2e, are well represented by the calculated isointensity surfaces after scaling of the structures in Fig. 2b to exposure threshold of *I*_*th_scan*_ = 0.5. The lateral fall off of laser exposure yields ~100% filling fraction at the center *x* = 0 mm position (solid zone in Fig. 2d-ii), opening into dense and low density 3D bicontinuous structure at 2 mm offset (Fig. 2e-i) and 3 mm offset (Fig. 2e-ii) positions, respectively, that transitions into underexposed and washed out photoresist beyond ~3.5 mm lateral offset. The observed lateral and axial periodicities of *a* = 570 nm and *c* = 1200 nm, respectively, indicate a strong ~35% *c*-axis shrinkage that is commensurate with previous reports^18^,^27^,^47^. Single line scanning is therefore robust against pattern distortion from exposure zones with missing diffraction orders for such large beam diameter (10 mm) and exposure distance (1 mm glass).

The lateral intensity fall-off, forming a solid central strip sandwiched by a 3D-PC structure (Fig. 2d-ii), can be quantitatively represented in Fig. 3a-i (single scan) by evaluating the 3D isointensity surface produced with increasing isointensity threshold for various lateral offset positions from the beam center. An isointensity threshold of *I*_*th_scan*_≈0.5 was set to generate an optimally open, stable and bicontinuous 3D structure. Figure 3a-i (single scan) provides an assessment of the lateral uniformity (refer to Methods – Laser Scanning Holography) of the 3D structure expected for a single exposure scan by fixing the isointensity value across the full *x*-range. For the case of *I*_*th_scan*_ = 0.48 (dashed horizontal line in Fig. 3a-i), one anticipates the formation of bicontinuous 3D PC structure in combination with overexposed solid and underexposed photoresist in zones that match well with the observation in Fig. 2d,e. Here, a 3 mm wide solid zone was observed sandwiched by bicontinuous 3D PC structure of about 2 mm width and further sandwiched by underexposed (washed away) photoresist.

### Holographic interference lithography: Parallel scanning exposure

To improve the exposure uniformity and further extend the lateral fabrication dimension, parallel scanning exposure was considered by optimizing the scan-to-scan offset *d* as depicted in Fig. 3b. With a coarse scan-to-scan offset of *d* = 4 mm, representing a 40% overlap of the beam, the isointensity exposure window for forming open, bicontinuous 3D PC structures was found to oscillate laterally on 2 mm steps as shown in Fig. 3a-ii (*d* = 4 mm). This isointensity window oscillates between *I*_*th_scan*_ = 0.408 to 0.475 (at *x* = 1 and 3 mm) and *I*_*th_scan*_ = 0.495 to 0.600 (at *x* = 0, 2 and 4 mm) ranges. Hence, the optimum laser exposure for creating open bicontinuous 3D structure varies significantly by 87% relative to the isointensity window width, predicting formation of parallel over-and-under exposed PC strips on 2 mm steps with 4 mm period.

A further reduction of the scan-to-scan offset to *d* = 1.5 mm for 85% beam overlap dramatically improves in the predicted uniformity of the 3D PC structure as noted in Fig. 3a-iii (*d* = 1.5 mm). The optimum exposure for generating open bicontinuous PC structure now varies by 36.6% of the isointensity window, which oscillates between *I*_*th_scan*_ = 0.429 to 0.545 (i.e. *x* = 0.7 mm offset) and *I*_*th_scan*_ = 0.466 to 0.593 (*x* = 0 and 1.5 mm offsets). Hence, a 15% beam scanning offset promises to form a continuously open and bicontinuous 3D PC structure at all lateral beam positions with *I*_*th_scan*_ set in the range of 0.466 to 0.541, but with a varying filling fraction that oscillates on 1.5 mm period.

A wide opening of the isointensity window across all lateral positions was found for scan-to-scan offset of *d* = 1 mm as shown in Fig. 3a-iv (*d* = 1 mm), representing 90% overlap of the 10 mm beam diameter. The optimum laser exposure for generating bicontinuous structure now varies only ~15.4% of the isointensity window width in the range from *I*_*th_scan*_ = 0.435 to 0.554 (*x* = 0 or 1 mm) to *I*_*th_scan*_ = 0.448 to 0.572 (*x* = 0.5 mm). Hence, 3D bicontinuous structure is anticipated to form at all lateral positions, over the entire sample surface, with exposure in a wide *I*_*th_scan*_ range of 0.450 to 0.554. In this way, parallel-line scanning of top-hat beam shape across a phase mask promises to form highly contrasting 3D optical interference intensity patterns, and to generate a uniform bicontinuous 3D PC structure over large area. Further reduction of the beam offset (<10%) will offer a diminishing improvement of the PC uniformity, while demanding faster and less stable scanning. Hence, scan-to-scan offsets were set at 10% of the beam diameter for experimental study, with laser exposure tuned to match the ideal *I*_*th_scan*_ = 0.5 level as indicated (dash line) in Fig. 3a-iv (*d* = 1.0 mm).

To test the theoretical modeling, scanning laser exposure of photoresist with 1 mm glass exposure distance and 1 mm scan-to-scan offset of a 10 mm diameter beam (Fig. 3b) was found to form a large area 3D PC structure over the entire 15 mm × 15 mm phase mask area as seen in the optical image in Fig. 3c. SEM cross-sectional and top-view images as shown in Fig. 3d,e, respectively, revealed a high quality 3D BCT-like structure, having transverse and axial periods of *a* ≈ 560 nm and c ≈ 1200 nm, respectively, that are in good agreement with the isointensity surface simulations shown as insets in each figure. Therefore, laser scanning is shown to extend the fabrication area significantly beyond the laser beam size in contrast with the circular disk formed by static exposure (Fig. 2b) or the non-uniform strip formed by single scan exposure (Fig. 2d).

**Figure 4:**
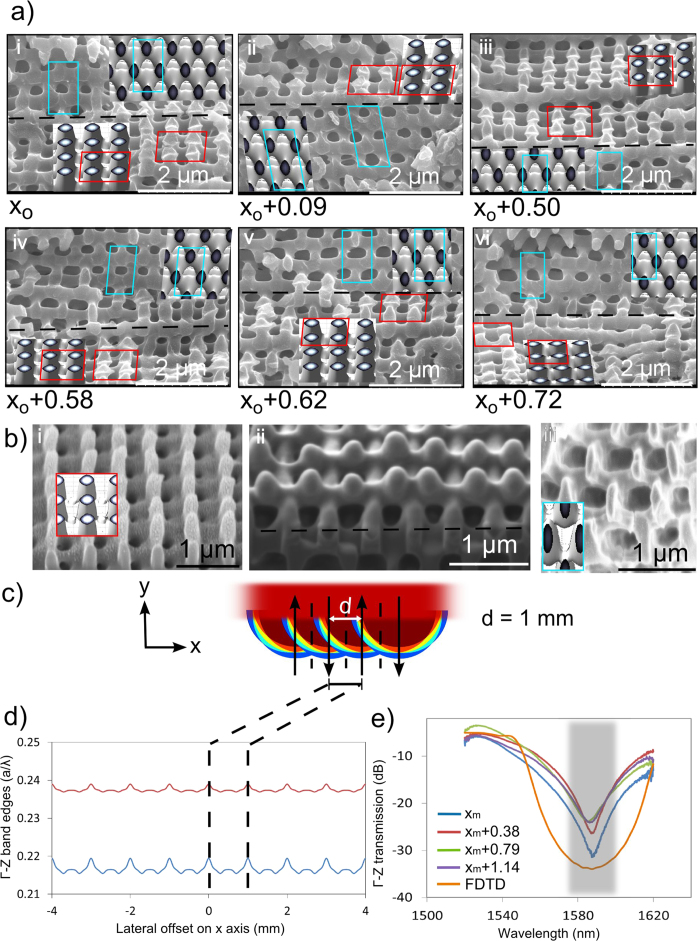
Microscopic and photonic band gap evaluation of the uniformity of 3D PC structure formed in photoresist by parallel scanning HIL with 10% lateral offset. (**a**) (i through vi) SEM images of bicontinuous 3D PC structure after cleaving and observed at ~35 degrees from various positions shifted laterally (*x*_*o*_ as labeled in mm), and matched with simulated intensity profiles on side and top facets (insets images). The red and cyan box pairs in each subset of (i) to (vi) denote matching areas on Γ-Z and Γ-X planes, respectively. The black dashed lines in (**a,b**) mark the boundaries between Γ-Z and Γ-X planes. The photoresist was formed by parallel scanning of 10 mm beam diameter with 10% offset separation of *d* = 1 mm. (**b**) Angled SEM views of a (i) top facet, (ii) corner and (iii) side facet of a PC structure following focused ion beam milling and their matching simulated 3D structures (insets). (**c**) The band edges of the 1^st^ photonic stop band along the Γ-Z direction calculated from isointensity patterns formed at positions shifted orthogonally to the scanning direction. (**d**) The Γ-Z directional transmission spectra recorded at various lateral positions (*x*_*m*_ shifts as labeled in mm) of HIL formed samples are well aligned. However, the 20 dB stopband width of 25 nm (dark band) falls short of the 50-nm width shown predicted by FDTD, possibly owing to nonuniform filling fractions and periodicity in the experimental sample.

The uniformity of the 3D microscopic structures formed over this extended area was more closely examined by cross-sectional SEM imaging and recorded transmission spectra of Γ-Z photonic band gaps (PBGs) along one cycle of the scan-to-scan offset (*d* = 1 mm) distance. PC templates were cleaved laterally to the laser scan direction (*x*) to record the sequence of SEM images shown in Fig. 4a at arbitrary offset positions of *x* = *x*_*o*_ to *x*_*o*_ + 0.72 mm. The tilted view (~35^0^) images simultaneously reveal structures for both horizontal planes (Γ-Z) and cross-sectional views (Γ-X). A clean cleavage of planes was difficult to form in the present sample, yielding the multi-terraced structures. Nevertheless, identical symmetry of BCT-like structure was recognized at all observed positions by a rectangular array of holes in photoresist on Γ-Z (horizontal) planes and a tetragonal array of holes in photoresist on Γ-X (front vertical) planes, with expected transverse and axial periods of *a* ≈ 560 nm and c ≈ 1200 nm, respectively, consistent with observations for the static and single-line scan exposures shown in Fig. 2. The facets in Fig. 4a (i-vi) cut through varying crystalline cross-sections and have been distorted during the mechanical cleaving to give an impression of varying filling fraction. Focused ion beam (FIB) milling was then applied to enable the higher resolution top (Fig. 4b-i), tilted (Fig. 4b-ii) and side (Fig. 4b-iii) view SEM images that confirmed formation of uniform periodic BCT structure with the expected *a* ≈ 560 nm and c ≈ 1200 nm. These images provided an estimated filling fraction of 0.60 ± 0.10 that may be connected with the isointensity surfaces expected for *I*_*th_scan*_ = 0.50 in Fig. 3a (bottom-right, *d* = 1.0 mm). The scanning exposure model also predicted a bicontinuous BCT-like structure of similar filling fraction, varying from 0.597 to 0.625 over the *x* = 0 to 1 mm lateral offset cycle.

A more definitive assessment of the 3D PC uniformity throughout the full photoresist thickness was made by recording the Γ-Z stop bands as a function of lateral offset positions (refer to Methods—Simulations and Spectral recording). Following first the *I*_*th_scan*_ = 0.5 exposure level in Fig. 3a (bottom right, *d* = 1 mm), the first order Γ-Z directional PBGs were calculated to provide the band edges shown in Fig. 4c over an 8 mm wide exposure zone. The band edges vary periodically (*d* = 1 mm) in a normalized wavelength range with minimum 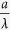 = 0.215 to 0.237 at *x* = 0.3 mm and 0.7 mm positions and of maximum 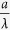 = 0.219 to 0.239 at *x* = 0 and 1 mm positions. We infer a median band gap position of 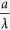
= 0.2273 (λ = 1592.4 nm scaling from 2507.7 nm for *a*=570 nm after 36.5% photoresist shrinkage was considered) that varies only ± 1.33% in the λ = 1579.2 to 1600.1 nm range. This high uniformity was verified experimentally by the nearly identical Γ-Z directional transmission spectra in Fig. 4c that were recorded over the lateral positions *x* = *x*_*m*_ to *x*_*m*_ + 1.14 mm that exceeds a full scan-to-scan offset cycle. Strong > 20 dB stop bands of ~25 nm width were observed at all lateral positions tested, yielding a peak wavelength centered at 1586.7 nm with only ± 0.1% variation. These observed mid-band wavelengths therefore match closely with the average of computed values, but do not sharply follow the predicted 1.33% lateral oscillations (Fig. 4b) due to limited spatial resolution (15 μm) of the probe beam, and variance in the photoresist chemistry and processing. The Γ-Z directional transmission of a matching BCT structure (32*c* = 38.4 μm thickness) was calculated by finite difference time domain (FDTD) method, providing good alignment to the experimental data but with a stronger (33 dB) and broader stopband as shown in Fig. 4d (orange line). The weaker observed stopbands arise here from small process variations inherent in the photoresist development (i.e. Fig. 4b-iii) and not from the small variation in lateral beam exposure (Fig. 4c). The 25 dB to 30 dB variation in stop band minima are inferred to arise from a high sensitivity to small changes expected in the filling fraction with lateral shifts. Overall, however, the parallel beam scanning method with 10% beam diameter overlap presents a low overall modulation of the bandgap as predicted in Fig. 4b and verified in Fig. 4c.

The present demonstration of scanning HIL was based on a non-ideal top-hat profile of the laser beam having ~15% intensity variation. Nevertheless, the intensity variation was effectively smoothed out by the multiple overlapping beam scans as evidenced by the narrow variation of the observed stopband spectra (Fig. 4c). The present beam overlapping and scanning approach is promising to extend similar accommodation to highly non-uniform intensity profiles such as verified for a Gaussian beam shape. Scanning HIL therefore facilitates a forgiving means for generating uniform 3D PC structure over ultra-large area that would be impossible in static exposure.

The results in Figs 2, 3, 4 confirm that the new approach of scanning laser holographic lithography can seamlessly stitch nanostructure together across multiple parallel laser scans and produce 3D periodic PC structure over large area. This seamless stitching is anticipated to scale to smaller beam diameters when beam scanning offsets are similarly scaled to ≤10% of the beam diameter and exposure distance is held at ≤10% of the beam diameter to suppress exposure from zones of incomplete diffraction orders (Fig. 1b). The prospects for writing highly uniform 3D PC structure in smaller, high resolution patterns are considered next.

### Flexible 3D direct writing of optofluidic components

The scaling down of the beam diameter in scanning HIL is evaluated for thick photoresist (40 μm) towards a new direction for flexible and monolithic fabrication of 3D PC structure integrated within an optofluidic laboratory on chip. This scaling was tested to the minimum laser beam diameter beyond which diffracted beams were no longer sufficiently overlapping to create highly contrasting 3D periodic interference patterns in the photoresist (refer to Supplementary Fig. S2). Scan velocity and laser power modulation was further applied to precisely control the isointensity exposure (refer to Supplementary Note 1) and thereby tune or chirp the 3D PC stopbands as well as to under or over expose the photoresist to create open and solid zones, respectively.

In order to accommodate the minimum possible laser beam diameter, photoresist was made to contact the phase mask, defining a maximum exposure distance of 40 μm inside the present SU-8 photoresist. By reducing the exposure distance from *z* = 1 mm to 40 μm for 10 mm beam diameter, the zone of five-beam interference increased dramatically from 77% to 99.0% areal overlap as seen in Supplementary Fig. S2a, in contrast with Fig. S2b (reproduced from Fig. 1b). Alternatively, this 25× smaller exposure distance (1–40 μm) offered a parallel opportunity to scale down the beam diameter by a similar factor of 10–400 μm without extending the narrow annular zone of incomplete diffraction orders seen in Fig. 1b. This beam scaling was verified experimentally, producing uniform 3D PC structure to 400 μm beam diameter, and then further tested at 200 μm and 60 μm where the five beam interference zone was predicted to shrink from 77% (400 μm diameter) to 56% (200 μm diameter) and 1% (60 μm diameter) as seen comparing Supplemental Fig. S2b, S2c, and S2d, respectively. One also expects the contrast of 3D periodic interference fringes to improve moving up to the top surface (*z* = 0 μm) of the photoresist, where beam overlap is the best in closest proximity with the phase mask surface.

**Figure 5:**
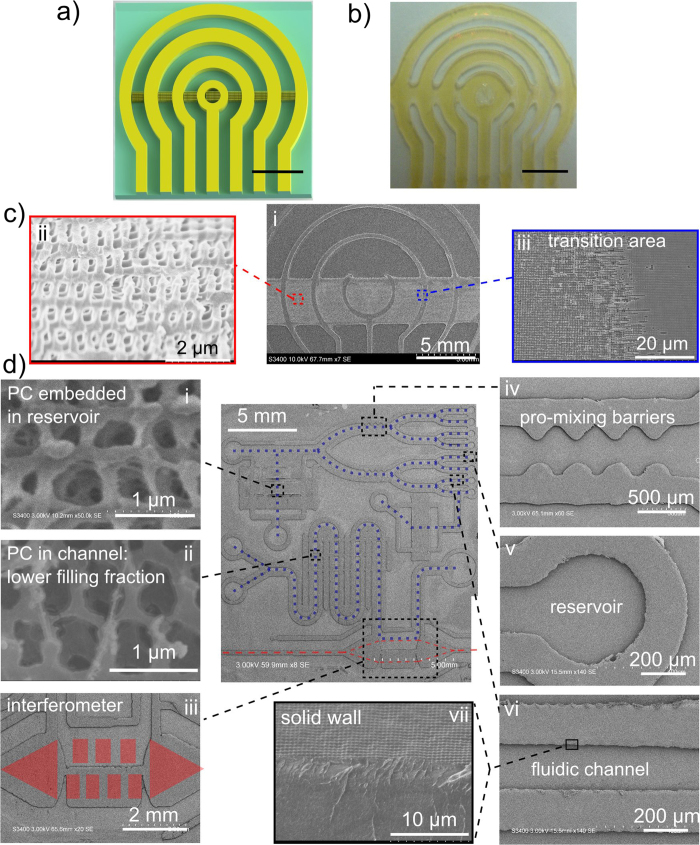
Demonstration of 3D PC embedded optofluidic devices and microsystems fabricated by direct-write scanning HIL. (**a**) A design of optofluidic device with open (unexposed) and 3D PC embedded (parallel 10% scan-to-scan offset) microfluidic channels laterally confined by solid photoresist (single line slow scan speed) and the corresponding (**b**) optical image of the fabricated device based on 2 mm beam exposure diameter and (**c**) higher resolution SEM images based on 200 μm beam diameter. SEM images of a (i) large area view and higher resolutions images reveal the (ii) bicontinuous 3D PC structure was formed against (iii) solid photoresist walls. The half arch shape in (**c–i**) indicates a partial delamination of the photoresist layer. (**d**) Demonstration of two optofluidic microsystems, laser written on a chip, integrating (i and ii) 3D PC structure with (iv, vi and vii) microfluidic channels and terminating in (v) reservoirs. (iii) A Mach-Zehnder interferometer (red shaded optical path) depicts a more functional optical component. The blue and red dashed lines indicate the fluidic and optical paths, respectively. The scale bars in (**a**,**b**) are 5 mm.

Laser direct writing with 2D patterning control of 3D nanostructures was first demonstrated for a 2 mm beam diameter to follow a circular channel design of solid and 3D PC lines as shown in Fig. 5a. The corresponding optical images of laser patterned photoresist in Fig. 5b were verified by SEM (images not shown) to have formed straight and curved solid lines of 2 mm width that defined the 1 to 3 mm wide open channels and reservoirs as per the design in Fig. 5a. With an increase in scan speed and the number of parallel offset scans (10 scans on Fig. 5b), 3D PC structure was verified to have formed, embedded in the channels against the solid walls. The device was patterned in a single step of laser scanning exposure, verifying the extension of HIL into a more flexible direct-write method for customized patterning of 3D PC embedded channels and reservoirs.

When further scaling of the beam diameter to 200 μm, an optimal laser exposure window for forming bicontinuous 3D PC structure was determined by creating a mesh of solid photoresist lines crossed with lines of 3D periodic structure with exposure varied by scan velocity (refer to Supplementary Fig. S3 and Note 1) while scanning with 10% beam diameter offset. The optical image in Supplementary Fig. S3a shows formation of a bicontinuous 3D PC structure in a narrow exposure window just above the threshold identified for photoresist development. The solid lines assisted in firmly bonding the 200 to 500 μm wide PC strips to the substrate. SEM images (not shown) confirmed the formation of bicontinuous 3D PC structure for all diameters down to 200 μm. This 200 μm diameter defined the resolution limit for the present case of 40 μm thick photoresist to yield 3D PC structure fully through the film thickness to the *z* = 40 μm exposure distance where the 5-beam interference zone in Supplementary Fig. S2c occupied 56% of the beam area. Hence, the scanning HIL method appears forgiving in generating highly contrasting 3D interference fringes to the limit when about one half of the exposure area is missing diffraction orders.

The 3D nanostructure was found to degrade significantly with further reduction of the beam diameter, as overall contribution from beam zones of incomplete diffraction orders became dominant, such as depicted in Supplement Fig. S2d for 60 μm beam diameter at 40 μm exposure distance. For the case of 30 μm beam diameter, a 3D periodic structure was no longer recognizable at the top surface of the photoresist as shown in the SEM images of Supplementary Fig. S3b. Here, a similar grid-scan exposure revealed formation of both solid (inset, left) and 3D porous (inset, right) structures that were controllable by the laser exposure, but prone to delamination at near-threshold exposure levels. The porous structure is highly distorted and does not show a clean periodicity in any direction. Hence, a minimum beam diameter limit of 200 μm was defined for formation of 3D PC structure in 40 μm thick photoresist. Further scaling to smaller beam diameter would be possible by scaling to smaller diffraction beam angles or to thinner photoresists, but with the disadvantage of creating fewer layers of the 3D periodic structure.

The direct-write approach in this limit of 200 μm beam diameter was tested to reproduce the design pattern of Fig. 5a, yielding the photoresist structure shown by the SEM image in Fig. 5c-i. A wide (~3.3 mm) band of bicontinuous 3D PC structure is seen at higher resolution in the 35^0^ tilted view of Fig. 5c-ii to have formed uniformly inside open channels that followed between the circularly shaped solid walls of ~300 μm width shown in Fig. 5c-i. The high resolution SEM image (Fig. 5c-ii) revealed an accurate reproduction of the expected BCT-like structure of *a* = 560 nm and *c* = 1200 nm periods as previously observed both in static (Fig. 2c) and scanning HIL (Figs 2e, 3d,e and 4a) exposures with the larger 10 mm diameter beam. Closer examination (blue box in Fig. 5c, middle) of the large-area 3D PC structure at the boundary with a solid line (Fig. 5c-iii) revealed a small 20 μm transition zone over which the bicontinuous 3D PC structure (left side) transformed into solid photoresist (right side).

For demonstration purposes, the flexibility of laser writing of an optofluidic chip (Fig. 5d) with channels embedded with 3D PC structure was examined for a different case of a Gaussian shaped beam with 200 μm diameter (full width at half maximum). A combination of writing solid walls (Fig. 5d-vii) in slow velocity (~1 mm/s) single scans and 3D PC templates (Fig 5d-i) in faster (~17 mm/s) parallel scans with 10% beam diameter offset led to creation of the two optofluidic microsystems that were laid out over the 25 mm × 25 mm area as shown in the center SEM image of Fig. 5d. The top system divides fluid flow into eight identical microchannels of 1500 μm × 200 μm cross-sectional area (Fig. 5d-vi) that terminate into 450 μm diameter reservoirs (Fig. 5d-v), while corrugated walls (Fig. 5d-iv) assist with fluid mixing. The blue dashed lines indicate the fluid delivery pathway. A large area (3 mm × 1.5 mm) of 3D periodic PC structure (Fig. 5d-i) was formed inside the rectangular reservoir to serve as a fine particle filter^30^ while the Γ-Z stop bands of the PC may be probed at normal incidence for refractive index sensing of the fluid^36^. The lower-left optofluidic system presents reservoirs that combine fluid flow into a single long serpentine mixing channel prior to optical analysis by an in-plane Mach-Zehnder (M-Z) interferometer^48^. The semi-transparent red dashed lines represent the optical path of the interferometer defined by the SU-8 photoresist waveguides (Fig. 5d, center), that expand and are collimated by the lenses (Fig. 5d-iii) to split into two parallel beam paths, one through the channel and one through solid photoresist. Such interferometers may serve as refractive index sensors of analytes that have potentially been filtered or separated by the upstream microfluidic channels. For example, the fine porous structure of the 3D PCs formed within the open serpentine channels (Fig. 5d-ii) may offer efficient separation of analytes during fluid flow. Such embedded 2D and 3D PC structures have proven useful as chemical mixers^38^, particle filters^32^, monodisperse microsieves^49^ and may improve molecular sieving or capillary chromatography columns with submicrometer size selectivity^50^ for advanced approaches in optofluidic integration on a chip^39^.

**Figure 6:**
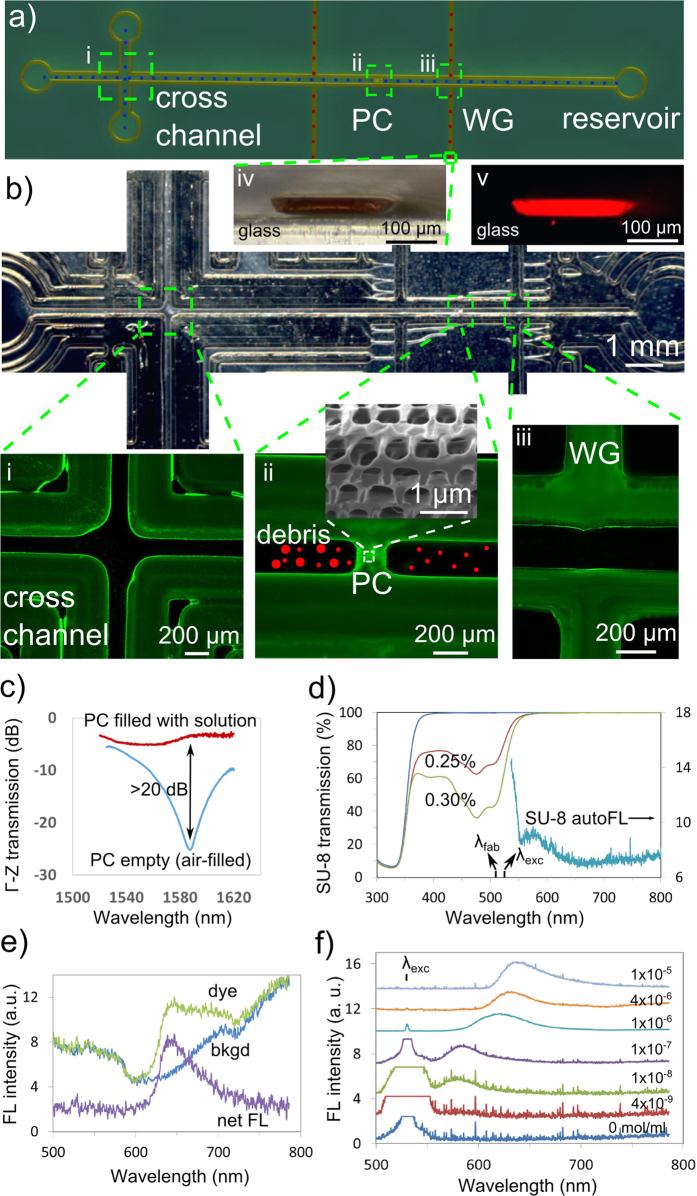
On-chip fluorescent excitation and detection with conventional cross-channel optofluidic chip. (**a**) The schematic of an optofluidic chip design with (i) crossed microfluidic channels (blue dotted lines), (ii) embedded 3D PC structure and (iii) integrated waveguides or WGs (red dotted lines). The optical microscope image of a scanning HIL fabricated chip is shown in (**b**), with the multi-wall channels arranged to fortify the platform for stable cover slip bonding. The (i) crossing channel, (ii) 3D PC structure (SEM in inset) and (iii) waveguide-channel intersection viewed with a fluorescent (FL) microscope. (iv) The waveguide end facet seen under (iv) optical microscopy and (v) red fluorescence imaging microscopy. (**c**) Γ-Z directional stopband transmission spectrum recorded before (air-filled) and after the PC structure in a sealed lab-on-chip was filled with Rhodamine B dye solution. (**d**) Optical transmission spectra of the fully developed SU-8 photoresist (40 μm thick) (exposure wavelength λ_fab_ = 514 nm) with 0 (blue), 0.25% (red) and 0.3% (green) photoinitiator (HNu-470) by weight together with the fluorescence spectrum (excitation wavelength λ_exc_ = 532 nm) for the 0.25% photoinitiator doped sample. (**e**) Fluorescence spectra recorded from the microfluidic channel filled with Rhodamine B dye solution of 2 × 10^−5^ mol/ml concentration (green) or water (blue), with laser excitation light (λ_exc_ = 532 nm) and fluorescent emission delivered by the respective incoming and outgoing on-chip multi-mode crossed waveguides. The difference spectrum (purple) presents the net dye-only fluorescence peaking at 635 nm. (**f**) Fluorescence spectra of Rhodamine B dye recorded from the same on-chip waveguide for decreasing concentration (as labeled) to a detection limit of 4 × 10^−9^ mol/ml.

The scope of novel opportunities was narrowed to fabricate an optofluidic chip that permitted integrated waveguide fluorescence interrogation of dye solution in microfluidic channels embedded with 3D PC structure. The layout followed the classical cross-channel architecture^51^ to define the optofluidic chip design as shown schematically in Fig. 6a, with the liquid flow channels labeled by the blue dotted lines and the two SU-8 polymer waveguides marked with the red dotted lines. A partial optical image of the fabricated device in Fig. 6b shows the main channel surrounded by multiple parallel walls that serve to fortify the platform for stable bonding with a poly(dimethylsiloxane) (PDMS)-coated cover slip. Fluorescence microscope images (Zeiss AxioObserver) show the cross-channel (Fig. 6b-i), a PC-embedded channel (Fig. 6b-ii), and a channel-waveguide crossing intersection (Fig. 6b-iii) under 505 nm wavelength band-pass filtering (Zeiss FITC 480 nm excitation filter set). In the inset of Fig. 6b-ii, an SEM image of the ion-milled PC structure reveals an open BCT-like structure as previously demonstrated (in Fig. 4). The narrow open pores serve to filter particles (red circles) in the solution exceeding ~500 nm diameter (particularly debris). In this way, the porous 3D nanostructure is desired in the optofluidic chip to improve signal-to-noise ratio and reproducibility in the fluorescent spectral recordings at very low dye concentration. The microscope image in Fig. 6b-iv and the fluorescent microscope image (Leica 560 nm Excitation filter set) in Fig. 6b-v show the SU-8 waveguide of Fig. 6b-iii terminating into a facet of 250 μm × 40 μm size. This large cross-section offers moderately efficient numerical apertures of ~1.25 and ~0.65 for respective lateral and vertical light collection at the channel-waveguide intersection.

A stopband sensing function of the 3D nanostructure is demonstrated in Fig. 6c where the strong ~25 dB stopband along the Γ-Z direction seen near 1.55 μm wavelength has become transparent (~4 dB) when filled with Rhodamine B dye solution. The optical transmission properties of the fully developed SU-8 photoresist are presented in Fig. 6d for 40 μm thick film (on a 1 mm glass substrate) with photoinitiator weight concentrations of 0% (blue), 0.25% (red) and 0.3% (green). The 0.25% concentration was selected to offer efficient acid catalyst generation without strong beam attenuation and thereby provide a uniform axial structuring at the laser exposure of *λ*_*fab*_ = 514 nm wavelength. This concentration further offered sufficient transmittance (88.5% in Fig. 6d) at the fluorescent excitation wavelength of *λ*_*exc*_ = 532 nm to permit sufficient light guiding over short waveguide lengths to excite Rhodamine B dye (Sigma-Aldrich) in the channel.

The SU-8 photoresist also presents an auto-fluorescent background spectrum peaking at ~580 nm with 532 nm excitation light as shown (aqua) in Fig. 6c, which is preferentially attenuated by the SU-8 waveguide over the desired Rhodamine B fluorescence peaking around 635 nm as shown (green) in Figure 6e for 2 × 10^−5^ mol/ml concentration. In this spectrum (Refer to Methods – Fluorescent integrated detection), the Rhodamine B dye solution was excited with 532 nm waveguide light, launched into the microfluidic channel of Fig. 6b-iii. The channel was sealed with a PDMS-coated cover slip and fluorescence was collected by a second SU-8 waveguide coupled via optical fiber to an intensified CCD (ICCD) spectrometer (Andor, AQ-6315A). This 635 nm red fluorescence was guided with high transmittance (i.e. ~100% (red) in Fig. 6d) through the SU-8 waveguide, with only a small component of SU-8 autofluorescent noise as seen (Fig. 6e) in the background spectrum (blue) for a pure water filled channel. The difference spectrum provided the net dye-only fluorescent response (purple) peaking at 635 nm as shown in Fig. 6e.

To examine the detection limit of the sealed-chip design, Rhodamine B dye concentrations were systematically reduced from 1 × 10^−5^ mol/ml (light blue) to 4 × 10^−9^ mol/ml (dark red) concentration as shown in Fig. 6f, yielding the sequence of weaker fluorescent bands that shift from 635 nm to 575 nm, respectively. This concentration based wavelength shift is well known for Rhodamine-type dye^52^. At the limit of detection (red) at 4 × 10^−9^ mol/ml concentration, the fluorescence has become significantly overshadowed by the excitation laser at 532 nm. For comparison purposes, this detection limit has been similar to the limit obtained with an external polarizer filter (i.e. 1 × 10^−9^ mol/ml concentration)^53^. While microscope-based sensing methods offer better detection limits (i.e. 2.5 × 10^−11^ mol/ml concentration) for lab-on-chip systems^54^, the on-chip waveguide excitation and detection promises significant miniaturization advantages that can bypass the bulky microscope optics as noted in related photoresist-based chip design^48^,^55^.

## Discussion and Conclusion

Compared with previous approaches of HIL with stationary beams^7^,^17^,^18^,^19^,^20^,^21^,^22^,^23^,^24^,^25^,^26^,^27^,^28^,^29^,^30^,^31^,^32^,^33^,^34^,^35^,^36^,^37^,^38^,^39^,^40^,^41^,^42^,^43^, both top-hat and nonuniform Gaussian beam can be used to form uniform 3D structures in laser scan HIL. Further, scanning an undersized exposure beam over a larger mask area until the full mask has been uniformly and completely exposed has been demonstrated such as the linearly shaped beams found in laser projection lithography^56^. In these cases, small linear beams have been preferred than full-wafer-size beams. Further, small diameter beam (e.g. 200 μm) permits direct writing of diverse optofluidic devices with a resolution similar to the beam size. These multi-component microsystems were exposed in one laser scanning step, offering up to ~5,600 individual points of exposure control for patterning over the present 15 mm × 15 mm phase mask area. Highly flexible designs have been demonstrated (Fig. 5) from a single, uniform and reusable phase mask that was found to be robust and precise on repeated laser exposure with many varied pattern designs.

The scanning holographic interference lithography method was demonstrated here (Figs 3 and 4) to offer formation of uniform 3D PC structure over large area. This uniformity was preserved for beam sizes scaled to a small as 200 μm size for 3D structuring inside thick (40 μm) photoresist. The method was forgiving in generating well contrasting interference patterns on which 3D PC structure could be formed (Fig. 5c–ii and Supplementary Fig. S3a) when up to one-half of the beam exposure area was missing diffraction orders at the deepest exposure distance (Supplementary Fig. S2c). The benefit of forming uniform 3D nanostructure also applies to non-uniform beams, as demonstrated by scanning HIL with a Gaussian beam shape that produced the bicontinuous 3D PC structure in Fig. 5d. The beam scanning approach in HIL was further extended to create patterns of solid, open and 3D PC structure (Fig. 5b,c) that enabled flexible fabrication of microfluidic, optical and PC devices on a monolithic optofluidic platform (Fig. 5d). However, this direct-write HIL approach would not be able to provide the same level of flexibility as two-photon direct-write does (as shown in Supplementary Fig. S1). In Fig. 6, novel opportunities were demonstrated on an optofluidic chip that permitted integrated waveguide fluorescence interrogation of dye solution in microfluidic channels embedded with 3D PC structure. A fluorescent detection limit of 4 × 10^−9^ mol/ml was demonstrated in Rhodamine B dye solution with neither use of bulky microscope optics nor external excitation light filter set. For some of the future directions, the spectrum of excitation light could be tuned to more transparent wavelengths and optimized to offer higher efficiency. Further, the flexibility of laser scanning exposure process may permit improved collection efficiency, e.g. by forming lens-shaped facets to the waveguide to effectively improve the numerical aperture. More elaborate testing of PC-embedded microfluidics could be designed for PBG-shift sensing applications. This direct-write approach offered rapid and well-controlled exposure levels in a single-step laser exposure of a single phase mask, opening a direction for customized fabrication of PC-embedded optofluidic systems. Overall, our technique promises significant miniaturization advantages and rich functionalities including particle filtering, waveguide fluorescent detection and PBG spectral sensing that could be harnessed in a single optofluidic chip.

The present scanning method should extend readily to other phase mask designs that control the 3D nanostructure symmetry and motif and form, for example, TTR^17^, woodpile^18^,^19^ to diamond-like^20^,^21^,^22^,^35^ 3D PC templates, offering isotropic directional stopbands^33^,^34^ or monodisperse nanoporosity^49^. The prospects for embedding finer structures such as localized defects to form optical waveguides and cavities within the present 3D photonic crystal network may be further explored with a second direct writing step^15^,^40^ to add functionality such as with photonic, surface plasmonic and metamaterials integrated devices. The scanning 3D HIL may extend to other photosensitive materials, excited by single or multi-photons, such as photopolymerizable resins^57^, CdS-polymer nanocomposites^58^, and chalcogenide glasses^59^.

The direct writing speed demonstrated here is ~10^5^ μm^3^/s for 200 μm beam size and ~10^8^ μm^3^/s for 10 mm beam size in direct-write holography, which greatly exceeds the very slow writing rate of <10^3^ μm^3^/s^4^,^60^ for direct-writing by two photon processing. Alternatively, the hybrid method was able to form the 3D nanostructures (40 μm thickness) with areal throughput of ~10^7^ to ~10^10^ μm^2^/h for the respective 200 μm and 10 mm beam sizes, falling within the areal speeds reported in Supplementary Fig. S1. This hybrid method of direct-write and large area processing offers an exposure rate of ~4 × 10^8^ μm^3^/s per watt in 40 μm thick resist that scales to high processing speed of ~9 wafers per minute (30 cm diameter) with an industry-scale laser of 1 kW power. However, scanning HIL fabrication is currently limited to the 15 mm × 15 mm phase mask area and would require development of larger area masks or new exposure approaches based on wafer-type step-and-repeat or cylindrical roller^61^ type phase masks.

In conclusion, we have introduced a laser scanning holographic method for phase mask lithography that demonstrated improved 3D structure uniformity over large area despite the detrimental contribution from interference of incomplete diffraction orders and non-uniform incident beams including Gaussian beam. The beam diameter was further introduced as a direct-write processing parameter for overall benefits of increased fabrication throughput and structural flexibility, filling the unexplored gap between two-photon direct writing and static HIL (refer to Supplementary Fig. S1). Scaling of beam diameter to small sizes has permitted direct-write patterning of multi-functional microsystems on a chip with up to 5,600 points of exposure control over 15 mm × 15 mm area. By combining the advantages of HIL and direct writing, the scanning HIL method presented a rapid and well-controlled patterning method for novel and flexible integration of diverse optofluidic components by means of velocity and power modulation. The results underpin the promise of future advances in multiple science and technology fields encompassing nanotechnology and the large scale integration of photonic, microfluidic, biological and chemical devices that can be applied in novel diagnostic, processing and lab-on-a-chip microsystems.

## Methods

### Photoresist preparation and development

Mixtures of SU-8 series photoresist (MicroChem, SU-8 2050 and 3050) with 0.25% to 0.3% weight HNu-470 (Spectra Group Ltd.) photo-initiator and 2.5% OPPI co-initiator (Spectra Group Ltd.) were spin coated onto 25 mm × 25 mm glass substrates at 3000 rpm for 50 s, resulting in approximately 40 μm thick photoresist with refractive index of *n*_*r*_ ≈ 1.6. Prior to and after laser exposure (details below), the photoresist sample was both soft-baked and post-baked over a temperature ramp of 65 °C and 95 °C in a period of 1 to 10 min. Following post baking, the photoresist sample was immersed in SU-8 developer (MicroChem) for 6 to 10 min at room temperature. A 0.3% photo-initiator concentration in 40-μm thick photoresist was measured to provide ~2% and ~12% optical absorption prior to and after development, respectively. These values exclude the Fresnel reflection losses and are too small to cause measureable (10 nm) decrease in nanostructure sizes from the top to the bottom of the present photoresist films.

### Laser scanning holography

The 514.5 nm wavelength output of an Argon ion laser (Coherent, Innova Sabre MotoFred, 3 watts) was converted to circular polarization, spatially filtered and expanded to a collimated Gaussian beam in a range of 12.5 to 25 mm diameter (full width at half maximum). An iris of variable (2–10 mm) diameter selected a nearly top-hat beam profile of 1.27 ~ 1.59 W/cm^2^ intensity (with ~15% variation) that was made normally incident onto a 2D binary phase mask (fused silica) of square grid pattern having *Λ*_*x*_ = *Λ*_*y*_ = 570 nm period and 600 nm grating depth (Ibsen, Netherland) as shown in Fig. 1a. The binary phase mask generated 1^st^ order diffraction angles of 65.1^0^ in air and 34.34^0^ in the photoresist. In this configuration, a 10 mm beam diameter and an exposure distance of *z* = 1 mm (photoresist to phase mask surface) led to ~0.8 mm offsetting of the four 1^st^ order beams from the 0^th^ order beams as indicated in Fig. 1b. Figure 2b considers how the intensity pattern for this exposure condition will accumulate when scanning the beam. The intensity patterns will vary significantly with offset positions, shown for *x* = 0, 2.2, 4.4 and 5.5 mm (dashed line), where the net intensity pattern was include the 1-beam (light blue), 2-beam (cyan), 3-beam (yellow), 4-beam (orange), 5-beam (dark red), and then reversely, 4-beam, 3-beam, 2-beam and 1-beam zones in the case of the center beam position at *x* = 0 mm.

To determine the bicontinuous structure processing window for single-scan exposure, we examine the figure shown in Fig. 3a-i. At the beam center (*x* = 0 mm), a high isointensity value of I_th_scan_ > 0.60, marked in green, marks an insufficient laser exposure to create a connected 3D structure, leading to washing away of all photoresist during development. Alternatively, the blue region of I_th_scan_ < 0.50 represents overly exposed photoresist where open parts of the desired BCT-like structure have closed to prevent penetration of the developer. One then finds a narrow exposure window of isointensity I_th_scan_ = 0.50 to 0.60 for creating bicontinuous open 3D PC structure in the central beam position (x = 0 mm). In moving away from the beam center (|*x*| > 0), this bicontinuous window narrows and shifts to lower isointensity values, reaching I_th_scan_ = 0.41 to 0.49 at the *x* = ± 4 mm positions, beyond which an unrecognizable structure will form due to multiple missing diffraction orders near the *x* = ± 5 mm beam edge.

Alternatively, various convex lenses were used to image the top-hat laser profile to a reduced beam diameter of 2 mm to 200 μm range onto the phase mask. The unmasked Gaussian beam was also directly focused by similar lenses to provide Gaussian beam profiles of 30 μm to 300 μm. Smaller areal interference structures of ~5 μm size have previously been demonstrated in the case of thinner photoresist^62^. In the cases of smaller beam diameter (<1 mm), the photoresist was gently positioned in direct contact (*z* = 0 mm) with the phase mask to ensure optimal exposure of fully overlapping orders (Fig. S2a) to interfere within the resist thickness. Exposure windows for forming bicontinuous PC and solid structure were then tested in static, single-line scanning, and parallel scanning delivery modes for variable power levels and scan speeds as described in Supplementary Note 1 and Fig. S3. The paired phase mask and photoresist were scanned against a fixed laser beam with an Aerotech stage (ABL10150 model) of 2 nm resolution and 200 nm position repeatability. Example exposure levels include: 1 to 1.25 W power at 10 mm mm beam diameter to generate solid lines, single-line PC and parallel scanning PC structures (10% beam diameter offset) at ~0.2, ~0.45 and ~3 mm/s scanning speeds, respectively, for SU-8 2050 photoresist; 50 ~ 80 mW power at 200 μm beam diameter to generate solid lines, single-line PC and parallel scanning PC structures (10% beam diameter offset) at ~1, ~3.5 and >17 mm/s scanning speeds, respectively, in SU-8 3050. A lab-on-a-chip system such as presented in Figs 4, 5, 6 required exposure times varying from 20 s to 20 min. The photoresist sample was readily separated from the phase mask after laser exposure.

Diffraction from the iris aperture introduced a small distortion of the beam shape on the phase mask, forming fringes in a ~100 μm wide outer annulus for the case 2 mm or larger beam diameter. In this case, >90% areal uniformity was maintained on the phase mask. Diffraction effects became more significant to create non-uniform beam illumination when focusing to smaller beam size (<0.5 mm), but this distortion was largely washed out by the beam-scanning approach. Similar benefits were found with the Gaussian beam exposure on the phase mask.

### Simulations

Interference patterns and isointensity surfaces were calculated with a multiple beam interference model^23^,^24^,^35^ for relative diffraction efficiencies of 50% for the 0^th^ order and 12.5% for each of the four 1^st^ order beams generated by the present phase mask at 514.5 nm. Varying levels of threshold exposure were considered in the calculation of 3D isointensity surfaces in the photoresist, which in turn were applied into the plane wave expansion method^63^ to calculate the photonic band edges for the expected simulated BCT-like structures.

### Spectral recording of stopbands

The infrared output from a fiber source (Thorlabs ASE-FL7002, 1530–1610 nm) was focused with a 30× objective lens of 0.4 NA (New Focus) to a beam waist of ~15 μm diameter and ~1 mm depth of focus and aligned normally to the 3D patterned photoresist (40 μm thickness). The transmitted light was collected into a single-mode fiber and delivered to an optical spectrum analyzer (OSA, Ando6317B) set to 0.2 nm resolution. The transmission spectrum was normalized to a reference spectrum recorded through a non-patterned solid photoresist film of similar 40 μm thickness.

### Integrated fluorescent detection

The output beam of a 532 nm diode laser (Lasermate, 20 mW) was focused by an objective lens (10×, N.A. = 0.25) into a 50 μm single-core multi-mode fiber. The fiber output was butt-coupled to a SU-8 waveguide of 0.2 to 1 mm length, integrated into a laser-written lab-on-a-chip platform, and assisted by index matching oil (*n*_*oil*_ = 1.515) for improved optical coupling efficiency. Dye (Rhodamine B, Sigma-Aldrich) fluorescence excited in a microfluidic channel was collected by a second SU-8 waveguide of 3 to 10 mm length, and butt-coupled to an identical single-core multi-mode fiber with index matching oil to be evaluated by a fiber-imaging spectrometer with an intensified CCD (ICCD) detector (Andor, AQ-6315A).

To assess background noise, the auto-fluorescence spectrum of a fully developed SU-8 film was collected in free space in the backwards 45^0^ angle when excited by normal incidence 532 nm wavelength light. Laser-patterned SU-8 film was bonded to a glass cover slip (Corning #2845-25, 160 μm thickness) having a spin-coated diluted poly(dimethylsiloxane) (PDMS) coating of a few micrometer thickness to define a sealed optofluidic biochip with multimode waveguides (SU-8) intersecting the sealed channels. Rhodamine B (Sigma-Aldrich) of various concentrations was dissolved in DI water and pumped with 12 kPa pressure to fill the microfluidic channels. Hydrophilicity of the channel walls was induced by pre-flushing with Ethylene Glycol.

## Additional Information

**How to cite this article**: Yuan, L. and Herman, P. R. Laser Scanning Holographic Lithography for Flexible 3D Fabrication of Multi-Scale Integrated Nano-structures and Optical Biosensors. *Sci. Rep.* **6**, 22294; doi: 10.1038/srep22294 (2016).

## Supplementary Material

Supplementary Information
