# Supplementary material for: Laser Scanning Holographic Lithography for Flexible 3D Fabrication of Multi-Scale Integrated Nano-structures and Optical Biosensors
Source: Sci Rep. 2016 Feb 29;6:22294. doi: 10.1038/srep22294 (PMC4770283; doi:10.1038/srep22294)
Supplement: Supplementary Information [file srep22294-s1.pdf]

Laser Scanning Holographic Lithography for Flexible 3D Fabrication of Multi-Scale Integrated Nano-structures and Optical Biosensors

Supplementary Information

Liang (Leon) Yuan[1], Peter R. Herman[1]

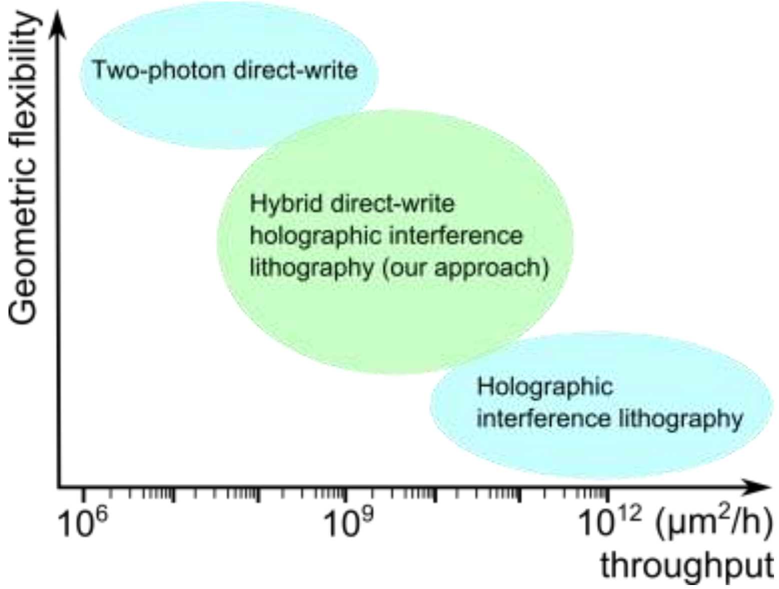

Supplementary Figure S1. A graphical representation highlighting the new nanofabrication domain of hybrid direct-write holography relative to the traditional processing domains of two-photon direct-write and holographic interference lithography (HIL). Two-photon direct writing provides a highly flexible means for high-resolution formation 3D structures, but typically limited to very slow rates of fabrication throughput (10^6-10^8 µm^2/s). In contrast, static holographic lithography offers exceptionally high throughput (10^{11}-10^{13} µm^2/s), but with limited flexibility in shaping the structures. This paper introduces the hybrid approach of direct-write holographic interference lithography that trades advantages between the flexibility of two-photon writing and the high throughput HIL. By scaling the exposure laser beam diameter from mm to µm sizes, 3D nanostructures of flexible shape can be formed over large area at areal fabrication rates in the range of 10^8 to 10^{11} µm^2/s. Throughput rates are adapted from Ref [1].

[1] The Edward S. Rogers Sr. Department of Electrical and Computer Engineering and Institute for Optical Sciences, University of Toronto, 10 King's College Road, Toronto, Ontario, M5S 3G4, Canada. Correspondence and requests for materials should be addressed to L. Y. (leon.yuan@mail.utoronto.ca).

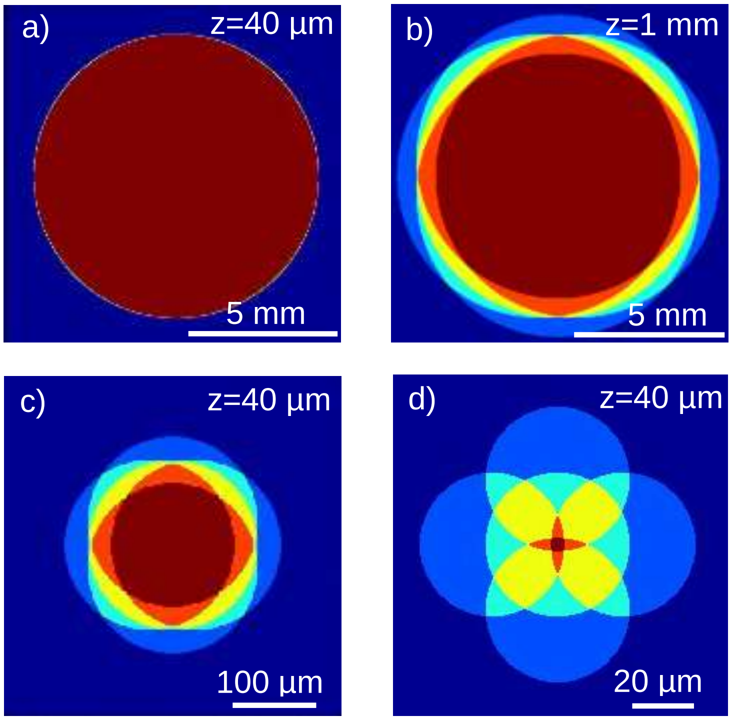

Supplementary Figure S2. Calculated positions of diffracted beams identifying exposures zones with overlap of all and partial number of diffraction orders for various beam diameters and working distances (*z*) from the phase mask. For 10 mm beam diameter, all diffraction orders strongly overlap at (a) *z*=40 µm exposure distance (99%) but (b) separate to yield 77% areal overlap at *z*=1 mm distance from the phase mask. Scaling to smaller beam diameter, the areal beam overlap of all diffraction orders for *z*=40 µm exposure distance reduces to 56% and 1% for (c) 200 µm and (d) 60 µm beam diameters, respectively. The color coding of zones with 5-beam (dark red), 4-beam (orange), 3-beam (yellow), 2-beam (cyan) and 1-beam (light blue) overlap is adapted from Fig. 1 in the main text.

Supplementary Note 1.

One significant objective in developing the laser scanning method of holographic interference lithography (HIL) was determining the highest writing resolution possible in thick photoresist (40 µm) as the laser beam diameter was scaled down in size. A systematic procedure of writing grid lines at varying exposure conditions was followed as beam diameter was reduced from 2 mm to 30 µm to confirm formation of a porous, uniform, and bicontinuous photonic crystal structure that bonded well with the substrate. Contrasting examples of 200 µm and 30 µm beam diameters are summarized here.

Figure S3*a* and *b* show respective optical and SEM images of mesh patterns produced under scanning beams of 80 mW power at 200 µm diameter and 8 mW power at 30 µm diameter, respectively. To improve adhesion, a grid of crossing lines were formed with slow velocities, shown in the vertical axis, to cross and lock down the parallel overlapping exposures recorded horizontally at lower exposure (i.e. high velocity). The line exposures were varied systematically with changing scanning velocity as labeled in Fig. S3*a*, and examined by optical microscopy as also shown in Fig. S3*a* to identify the threshold exposure for photoresist solidification and adhesion. The formation of bicontinuous 3D photonic crystal was examined in the band of parallel scanning exposures of 20 lines offset by the

10% beam diameter as found optimal in generating uniform stop bands (Section 4). The PC structure was verified by SEM imaging (not shown) to mark the narrow laser exposure window labeled as 'PC' in Fig. S3*a* for 200 µm beam diameter, which was separated by 'underexposed' and 'overexposed' zones also identified. The threshold for forming single-scan solid (vertical) lines within the mesh design were seen at scan speeds slower than 1.8 mm/s, but speeds slower than 1.2 mm/s were preferred for more reliable substrate bonding. The formation of bicontinuous PC templates were initiated at speed of 18 mm/s, while lower exposures at 25 mm/s also proved sufficient when stabilized by the closely positioned crossed (vertical) lines. The optimized exposure conditions of around 15 mm/s and 50 mW were then applied in the demonstration of the optofluidic microsystem in Fig. 5, where bicontinuous 3D PC structure is seen in the SEM images of Fig. 5*c* and *d*.

With a further reduction in beam diameter to 30 µm, a similar velocity varying pattern of single exposure lines crossed by bands of 20 exposure lines (10% offset) revealed the formation of solid photoresist as verified in the SEM image of Fig. S3*b*. However, delamination is more prominent at the threshold exposure conditions here due to the narrower contacting surface of this smaller diameter beam. The ~30 µm wide solid lines first appeared at a threshold scan velocity of 2.4 mm/s, but higher exposures of less than 1.6 mm/s scan speed offered more reliable bonding to the substrate. Higher resolution examination of the horizontal bands formed by parallel scans were found at a threshold exposure of 28 mm/s scan speed, but yielded only a random 3D porous structure as shown in the inset SEM image of Fig. S3*b* (upper-right). The open structures do not follow the expected periods (Λ_x=Λ_y=570 nm) produced by the phase mask interference, as further discussed in the main article. The formation of this random 3D structure existed in a very narrow exposure window, labelled as 'porous' in Fig. S3*b*, before giving way to formation of solid structure with higher exposures, as verified by the inset SEM image in Fig. S3*b* (upper-left).

Beam diameters between 30 and 200 µm were also examined, providing only random or distorted 3D nanostructures, such as the random porous structure reported for 40 µm diameter (Fig. S3*b* upper-right inset). The onset for creating well ordered periodic 3D nanostructure was therefore found at 200 µm beam diameter for the present case of proximity exposure of 40 µm thick photoresist with a 570-µm period phase mask.

In conclusion, the formation of mesh lines by laser scanning 3D direct-writing holography revealed a narrow process window for writing lines of solid structure and bicontinuous 3D PC nanostructure. In the limit of smallest 200 µm beam diameter, a 80 mW power exposure provided solid lines below 1-2 mm/s scan speed for single lines and bicontinuous 3D PC structure at 10-25 mm/s scan speed for parallel scans (10% diameter offset). These exposure conditions were adopted for writing various patterned designs of optofluidic devices as shown in Fig. 5*d*, providing a single-exposure method for monolithic integration of diverse components within a single chip.

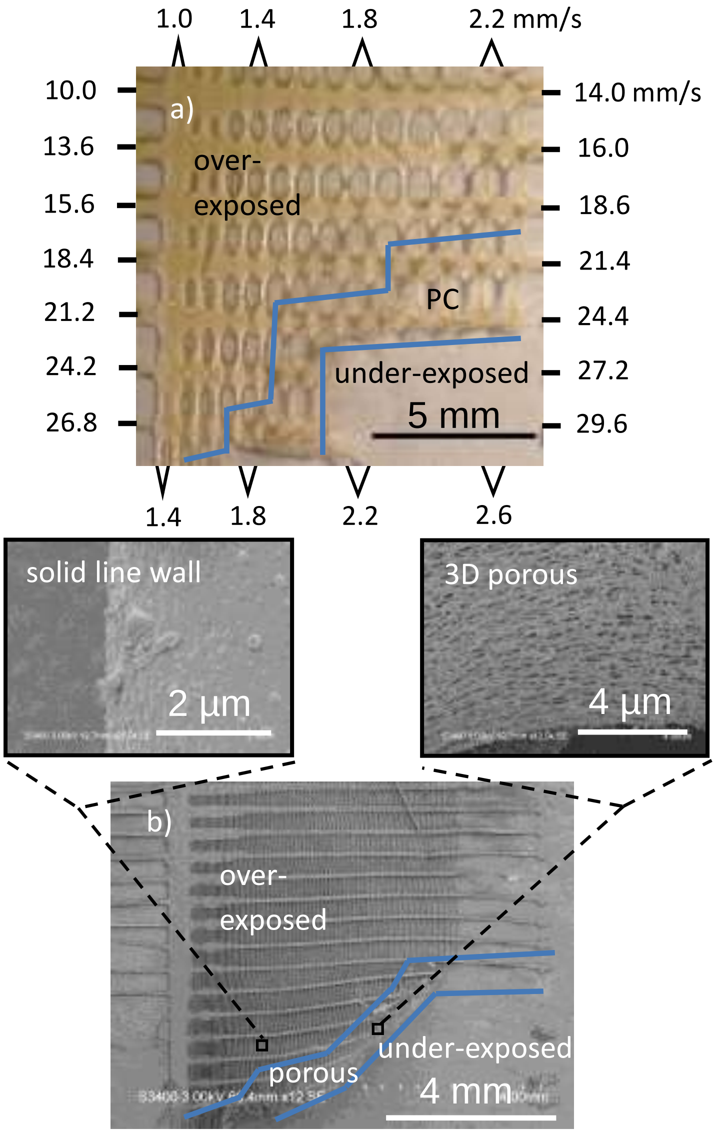

Supplementary Figure S3. Mesh patterns generated in thick photoresist by direct-write laser holography that identify the exposure window for forming bicontinuous photonic crystal templates, comparing (a) 200 µm and (b) 30 µm laser beam diameters scanned at varying velocity and parallel offset. Low-velocity single line scans shown vertically are crossed horizontally with groups of parallel exposure scans, each made with 20 parallel lines that were offset by 10% of the beam diameter. The grid patterns were formed with (a) a 200 µm beam diameter of 80 mW laser power and (b) with a 30 µm beam diameter of 8 mW laser power as shown in optical microscopy and SEM images, respectively. The scanning velocity was varied systematically over start and end values as indicated in (a) in mm/s. Similar scan speed values were used in (b).
